# Infections in Children Admitted with Complicated Severe Acute Malnutrition in Niger

**DOI:** 10.1371/journal.pone.0068699

**Published:** 2013-07-17

**Authors:** Anne-Laure Page, Nathalie de Rekeneire, Sani Sayadi, Said Aberrane, Ann-Carole Janssens, Claire Rieux, Ali Djibo, Jean-Claude Manuguerra, Hubert Ducou-le-Pointe, Rebecca F. Grais, Myrto Schaefer, Philippe J. Guerin, Emmanuel Baron

**Affiliations:** 1 Epidemiology and Population Health, Epicentre, Paris, France; 2 Epidemiology and Population Health, Epicentre, Maradi, Niger; 3 Microbiology Laboratory, Centre Hospitalier Intercommunal, Créteil, France; 4 Direction Générale de la Santé, Ministry of Health, Niamey, Niger; 5 Internal Medicine, Niamey University, Niamey, Niger; 6 Laboratory for Urgent Response to Biological Threats, Institut Pasteur, Paris, France; 7 Department of Radiology, Armand Trousseau Hospital, Paris, France; 8 Medical Department, Médecins Sans Frontières, Sydney, Australia; 9 Centre for Tropical Medicine, Nuffield Department of Clinical Medicine, University of Oxford, CCVTM, Oxford, United Kingdom; Menzies School of Health Research, Australia

## Abstract

**Background:**

Although malnutrition affects thousands of children throughout the Sahel each year and predisposes them to infections, there is little data on the etiology of infections in these populations. We present a clinical and biological characterization of infections in hospitalized children with complicated severe acute malnutrition (SAM) in Maradi, Niger.

**Methods:**

Children with complicated SAM hospitalized in the intensive care unit of a therapeutic feeding center, with no antibiotics in the previous 7 days, were included. A clinical examination, blood, urine and stool cultures, and chest radiography were performed systematically on admission.

**Results:**

Among the 311 children included in the study, gastroenteritis was the most frequent clinical diagnosis on admission, followed by respiratory tract infections and malaria. Blood or urine culture was positive in 17% and 16% of cases, respectively, and 36% had abnormal chest radiography. Enterobacteria were sensitive to most antibiotics, except amoxicillin and cotrimoxazole. Twenty-nine (9%) children died, most frequently from sepsis. Clinical signs were poor indicators of infection and initial diagnoses correlated poorly with biologically or radiography-confirmed diagnoses.

**Conclusions:**

These data confirm the high level of infections and poor correlation with clinical signs in children with complicated SAM, and provide antibiotic resistance profiles from an area with limited microbiological data. These results contribute unique data to the ongoing debate on the use and choice of broad-spectrum antibiotics as first-line treatment in children with complicated SAM and reinforce the call for an update of international guidelines on management of complicated SAM based on more recent data.

## Introduction

Malnutrition plays a major role in over one-third of all child deaths worldwide [1], [2]. Since malnourished children are especially vulnerable to infections, this combination forms a vicious spiral in which each condition exacerbates the other [3].

Yet in many regions of the world with high rates of childhood mortality and malnutrition, there is little to no diagnostic facility and the most common pathogens infecting malnourished children have barely been characterized in terms of types, prevalence, antibiotic resistance profiles or clinical presentation. Consequently, case management is empiric, following World Health Organization (WHO) guidelines developed over 10 years ago based on limited data [4], [5]. In these guidelines, routine oral cotrimoxazole is recommended for children admitted with uncomplicated severe acute malnutrition (SAM), although amoxicillin is often preferred. Ampicillin and gentamicin are recommended for children with complicated SAM as first-line treatment. If the child fails to improve within 48 hours, second-line treatment (e.g. chloramphenicol or other appropriate antibiotics for specific infections) are recommended. The guidelines do not differentiate between types of SAM (marasmus or kwashiorkor) or presence of underlying conditions such as HIV/AIDS.

Most data on infections in malnourished children come from Southeast Asia or Eastern or Southern Africa. There has been very little information on infections in children with complicated SAM from the Sahel region, despite the heavy burden of malnutrition in this area [6]–[8]. The Sahel reports some of the highest child mortality rates in the world, with malnutrition an associated cause of more than one third of these child deaths [9]. Within this region, Niger has the highest malnutrition burden in Africa and the fifth largest globally [10]. As a first step towards filling the information gap on infections in children with complicated SAM and formulating better evidence-based treatment guidelines, we conducted a study on children presenting with SAM plus an acute medical condition requiring hospitalization. We sought to assess the case fraction, etiology and clinical characteristics of invasive bacterial infection, malaria, respiratory and urinary tract infection and infectious diarrhea among children admitted with complicated SAM in Niger. We also reported antibiotic sensitivity of the pathogens isolated, and clinical outcomes.

## Methods

### Ethics Statement

The procedures followed were in accordance with the ethical standards of the Helsinki Declaration. The National Ethics Committee of Niger and the Comité de Protection des Personnes, Ile de France XI, France, granted ethical approval. Written informed consents for study participation, and separately for HIV testing, were obtained from participants’ parents or legal guardians. All treatments were provided free of charge.

### Study Setting

The region of Maradi is located in the southern part of Niger and shares a border with Nigeria. The region has the highest rates of acute malnutrition in the country [11]. Each year, the decrease in food quantity and quality experienced in the months preceding the harvest (August to October) is associated with an increase in wasting among children accompanied by an annual seasonal peak in malaria in October and November.

Since 2001, Médecins Sans Frontières (MSF), in collaboration with the Ministry of Health (MoH), has been involved in large-scale programs to treat children with SAM in Niger through outpatient and inpatient malnutrition treatment centers. Children with SAM and a medical complication (complicated SAM) were admitted as inpatients in an intensive therapeutic feeding center (ITFC) located centrally in the Maradi region. The ITFC included an intensive care unit (ICU), with one nurse for every 10 children and one medical doctor for every 20 children, a transition unit with one nurse for every 20 to 30 children and a nutritional rehabilitation unit with one nutrition assistant for every 30 children. As malnutrition is seasonal, the number of beds varied during the year with a maximum of around 300 beds during the peak season. Children were admitted to the ICU or transition unit according to the severity of the medical complications, or to the nutritional rehabilitation unit for cases with mostly nutritional problems (ie. anorexia).

The study took place between October 2007 and July 2008, when the study ended prematurely, due to unexpected closure of the MSF program.

### Study Population

The study population comprised all children aged 6 to 59 months with complicated SAM admitted to the ICU or transition phase of the ITFC during the study period. Children were excluded from the study if they had been referred from an outpatient or other health facility or had received antibiotics in the previous seven days, as assessed by history. SAM was defined as weight-for-height (WfH) less than −3 z-score of the median WHO growth standards and/or MUAC <110 mm and/or bipedal edema [12], [13]. Complicated SAM was defined as SAM accompanied by anorexia and/or kwashiorkor with bilateral pitting edema and/or another severe condition (severe anemia, severe respiratory tract infection, malaria with signs of severity, other severe infections such as meningitis or sepsis, diarrhea with dehydration, lethargy or acute neurological disorders, sickle cell crisis).

### Inclusion and Clinical Management

Clinical data on admission, at day 3 and at discharge were recorded in a standardized case report form designed for the study. In the case of death, clinical data from the last examination before death along with detailed treatment information were captured. The cause of death was assessed by the medical doctor on call according to the last medical examination (prior to death), medical history and laboratory examinations. Every medical record, including the medical records of the children who died, was then reviewed by 2 medical doctors (the study research assistant and the principal investigator of the study).

Children were treated according to WHO therapeutic guidelines for complicated SAM [4], [5] adapted by MSF. Dietary treatment included 8 daily meals of F-75 milk for stabilization in intensive care or transition unit, followed by F-100 milk for nutritional rehabilitation in cases exiting intensive care prior to discharge. Amoxicillin was given systematically, or parenteral ceftriaxone in cases of suspected severe or complicated infectious syndrome, i.e. lower respiratory tract infection, meningitis, sepsis or prostration**.** Treatment was modified based on indications such as non-improvement of clinical condition and/or results of bacterial culture and antibiotic sensitivity testing. Depending on the type of infection suspected, cloxacillin (skin infection, severe pneumonia, *S. aureus* bacteremia) or ciprofloxacin (urinary tract infection, severe, explosive or persistent diarrhea >72 hours, bloody diarrhea, bacteremia with suspected gram negative bacteria) was added in case of treatment failure, based on lack of improvement or worsening of symptoms within 72 hours following treatment. Children with uncomplicated malaria diagnosed either by rapid test and/or smear microscopy were given oral artesunate and amodiaquine for 3 days. Children with severe or complicated malaria received arthemether IM and then artesunate-amodiaquine if their condition improved, for 7 days total.

Children were discharged from the ITFC if they fulfilled all the following criteria: adequate appetite and consumption of ready-to-use therapeutic food; rising weight curve; no severe edema; no fever or signs of infection or diarrhea; not under active treatment with injectable antibiotics; mother able to attend regular weekly outpatient sessions at the nearest nutritional outpatient center if needed. Patients testing HIV-positive were referred after discharge to the Maradi Regional Hospital for enrollment in the MoH HIV program.

### Specimen Collection

Blood and urine samples were systematically collected at admission before antibiotherapy, and feces samples collected within 48 hours after admission. Two blood samples for culture were collected within 20–30 minutes of each other. Urine was collected using a Foley catheter. Stools were collected in a non-sterile collection pot.

Nasal swabs were obtained from every fourth patient included in the study, irrespective of clinical signs. Nasal swabs were obtained by rubbing the humid swab in each nostril before inserting in the virological transport medium.

A lumbar puncture was performed if the child presented at least one neurological sign indicating involvement of the central nervous system (reduced level of consciousness, coma; convulsions; hypotonia or hypertonia; neck stiffness, bulging fontanel, kernig or brudzinski signs; febrile purpura).

### Microbiology Methods

A dedicated bacteriology laboratory was established in the Maradi Regional Hospital for the purpose of this study. One milliliter of each blood sample was inoculated into a blood culture bottle (SIGNAL Blood culture System, OXOID, UK). Gram staining results from positive blood cultures determined subsequent choice of chocolate agar, blood agar, nutrient and/or Hektoen medium for subculture. Ten microliters of urine were inoculated onto a CHROMagar Orientation plate (CHROMagar, France). Stools were inoculated on Hektoen, DCL, and Campylobacter medium. Additionally, Muller Kaufman liquid medium was inoculated with the stool suspension for *Salmonella* enrichment. After 24 h, the Muller Kaufman broth was subcultured onto a CHROMagar Salmonella plate.

Identification of bacterial isolates from blood, CSF, urine and stool was done according to standard methods, using biochemical tests and the API*®* system (bioMérieux, France), followed by agglutination into specific antisera. Antibiotic susceptibility was assessed using the Kirby Bayer disk diffusion method on Mueller-Hinton agar and interpreted following the 2007 recommendations of the Société Française de Microbiologie [14].

### Other Laboratory Methods

Immediate bedside testing included those for blood glucose (One Touch Ultra 2, Lifescan, Inc., USA) and hemoglobin levels (HemoCue Hb 301, HemoCue, Sweden), a urine dipstick (Combur Test, Roche, Germany), and an HRP-2 based rapid diagnostic test (RDT) for *P. falciparum* malaria (Paracheck, Orchid Biomedical, India).

From each child, thin and thick blood films were prepared, Giemsa-stained, and read by experienced laboratory technicians. At least 100 high power microscopic fields of the thin film were examined to exclude the diagnosis of malaria. Complete blood counts were performed at the Maradi hospital laboratory using an ABX Micros 60 system. Sickle cell disease was investigated by hemoglobin electrophoresis using the Hydragel K20 System (Sebia Electrophoresis, USA). HIV serology was performed using Determine HIV 1/2 (Abbott Laboratories, USA) as a screening test, followed by Genie II (Bio-Rad, France) if the first test was positive. According to the national guidelines, if the tests were discordant, both tests were repeated.

Stools were investigated for the presence of rotavirus using the Vikia Rota/Adeno rapid test (Biomérieux, France).

Electrolytes measurements were retrospectively done on frozen serum specimens at Hôpital Bichat, Paris. Nasal swabs were stored at −80°C and transported to the Laboratory for Urgent Response to Biological Threats, Pasteur Institute, Paris. Quantitative reverse-transcription polymerase chain reactions (RT-PCR) specific for Influenza A (M, H1 and H3 genes) and B viruses as well as Respiratory Syncytial A and B Viruses (RSV), were carried out using primers and probes developed by the French National Reference Center for Influenza with a LightCycler 480 instrument (Roche Diagnostics, Switzerland) and a SuperScript III Platinum OneStep RT-PCR kit (Invitrogen™, California) [15].

### Chest Radiography

Plain antero-posterior chest X-rays were performed systematically on admission using a computed radiography system, except when the general condition of the child did not allow. The digital images were interpreted by clinicians on site for use in clinical management and re-read at the Hôpital Armand Trousseau, Paris, France with moderate correlation (kappa = 0.49) (manuscript in preparation). The reference results were considered definitive and are presented here.

### Definitions

Fever was defined as an axillary temperature above 38°C and hypothermia as an axillary temperature below 35.5°C. Diarrhea was defined as at least three watery stools in less than 24 hours, with or without blood. Severe dehydration was defined as the presence of 2 or more of the following: lethargy, sunken eyes, drinks poorly or not able to drink and skin pinch goes back very slowly [16]. Fast breathing was defined as more than 50 breaths per minute for children below 1 year of age and more than 40 breaths per minute for children above 1 year of age [17]. Hyperleukocytosis was defined by a number of leukocytes/mL of blood greater than 19,500 for children under 1 year of age, 17,500 for children between 1 and 3 years of age and 15,500 for children above 3 years of age [18]. Severe anaemia was defined as a haemoglobin concentration below 7 g/dL [19], hyponatremia as a sodium concentration below 130 mmol/L and hypokalemia as a potassium concentration below 3.5 mmol/L.

Systemic inflammatory response syndrome (SIRS) was defined according to the International paediatric sepsis consensus conference [20]. Briefly, SIRS was defined by the presence of at least two of the following four criteria, one of which must be abnormal temperature or leukocyte count: core temperature of >38.5°C or <36°C; tachycardia (heart rate >180 beats/minute for children under 2 years of age, >140 for children over 2 years); respiratory rate (>34 breaths/minute for children under 2 years, >22 for children over 2 years); leukocytes count (<5,000/mm^3^ or >17,500/mm^3^ for children under 2 years old, <6,000/mm^3^ or >15,500/mm^3^ for children over 2 years old). Sepsis was defined as SIRS with an associated suspected or proven infection, defined as follows: positive blood culture for known pathogen, urinary tract infection (see below), pathogen isolated from the stool in patients with diarrhea, malaria, or abnormal X-ray.

Germs isolated from blood cultures were divided into 3 categories: known pathogens that included all bacteria known to cause infections; possible pathogens that included bacteria that could be either a cause of bacteraemia or contamination; and contaminants that comprised all other bacteria. A urinary tract infection was considered if bacteriuria with a single pathogen was greater or equal to 10^4^/mL (*E. coli*) or to 10^5^/mL (other bacteria) regardless of the number of leukocytes in the urine, or if bacteriuria was greater or equal to 10^3^/mL for *E. coli* or to 10^4^/mL for other bacteria in the presence of at least 10^4^ leukocytes/mL in the urine [21]. Malaria was defined as a positive thick and/or thin smear.

### Statistical Analysis

Double data entry was done using EpiData 3.1 (EpiData, Odense, Denmark), and data analysis using Stata® (College Station, Texas, USA).

For categorical variables, we compared proportions using the chi-square or Fischer exact test; for continuous variables, medians were compared using the Wilcoxon rank-sum test. Kappa coefficient was calculated to measure inter-reader correlation of chest X-ray.

## Results

**Figure 1:**
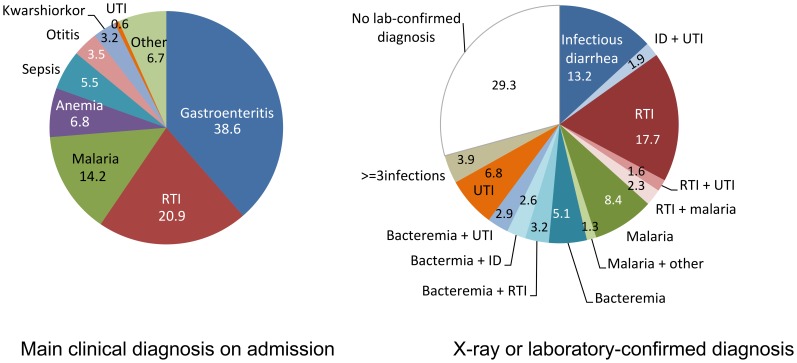
Main clinical diagnosis on admission (left) and laboratory or radiography-confirmed diagnosis (right) in the study population (N = 311).

A total of 311 children were included between November 19^th^ 2007 and July 22^nd^ 2008. Median age in the study population was 13 months (IQR: 10–24) and males represented 54.7% (n = 170/311). Forty-eight children (15.4%) presented with edema. The median WfH z-score was −3.8 (IQR: −3.3; −4.5) and the median MUAC was 116 mm (IQR: 108–122). Demographic, nutritional status and main diagnosis upon admission (gastroenteritis followed by respiratory tract infections, malaria and anemia) in the study population did not differ from the general population admitted to the ITFC during the study period (Table S1, Figure 1, Table 1).

**Table 1:** Socio-demographic, clinical and biological characteristics, overall and by type of infection.

		All (N = 311)		Bacteremia (N = 51)		RTI (N = 90)		Malaria (N = 44)		UTI (N = 48)		Infectious diarrhea (N = 62)		Sepsis (N = 111)		No confirmed infection (N = 51)	
		n	%	n	%	n	%	n	%	n	%	n	%	n	%	n	%
**Socio-demographic**																	
	Sex (male)	170	**54.7**	26	**51.0**	42	**46.7** *	21	**47.7**	20	**41.7**	32	**51.6**	51	**46.0**	34	**66.7**
	Age <12 months	127	**40.8**	16	**31.4**	33	**36.7**	14	**31.8**	28	**58.3** **	33	**53.2**	46	**41.4**	20	**39.2**
**Malnutrition**																	
	Oedema	48	**15.4**	11	**21.6**	8	**8.9**	10	**22.7**	7	**14.6**	5	**8.1**	13	**11.7**	12	**23.5**
	WfH z-score <4 (N = 263)	109	**41.4**	12	**30.0**	31	**37.8**	15	**44.1**	17	**41.5**	28	**49.1**	37	**37.8**	14	**35.9**
**Clinical signs**																	
	Fever	59	**19.0**	14	**27.5**	24	**26.7**	10	**22.7**	8	**16.7**	11	**17.7**	51	**46.0** ***	7	**13.7**
	Hypothermia	5	**1.6**	0	**0.0**	0	**0.0**	1	**2.3**	1	**2.1**	0	**0.0**	2	**1.8**	2	**3.9**
	Acute diarrhea	177	**56.9**	29	**56.9**	39	**43.3** ***	21	**47.7**	34	**70.8** *	NA	**NA**	56	**50.5**	28	**54.9**
	Severe dehydration	11	**3.5**	1	**2.0**	2	**2.2**	1	**2.3**	4	**8.3**	4	**6.5**	4	**3.6**	0	**0.0**
	Fast breathing	124	**39.9**	25	**49.0**	50	**55.6**	20	**45.5**	17	**35.4**	18	**29.0**	61	**55.0** ***	13	**25.5** *
	SIRS	149	**47.9**	28	**54.9**	50	**55.6**	22	**50.0**	22	**45.8**	23	**37.1**	NA	**NA**	21	**41.2**
	Lethargy	108	**34.7**	19	**37.3**	24	**26.7**	12	**27.3**	22	**45.8**	28	**45.2**	41	**36.9**	14	**27.5**
	Mycosis	68	**21.2**	18	**35.3** *	18	**20.0**	0	**0.0** ***	14	**29.2**	9	**14.5**	19	**17.1**	10	**19.6**
	Skin lesions	34	**10.9**	8	**15.7**	5	**5.6**	5	**11.4**	5	**10.4**	4	**5.6**	10	**9.0**	9	**17.7**
**Laboratory results**																	
	Positive blood culture (N = 302)	51	**16.9**	NA	**NA**	13	**14.6**	4	9.1	13	**27.7**	11	**18.0**	28	**25.7** **	NA	**NA**
	Sickle-cell disease (N = 278)	16	**5.8**	2	**4.4**	5	**5.9**	2	**4.7**	1	**2.3**	1	**1.8**	9	**8.9**	6	**13.6** *
	Severe anemia (N = 257)	50	**19.5**	11	**26.8**	15	**20.8**	13	**32.5**	6	**15.8**	7	**13.0**	22	**27.2** *	10	**22.7**
	Hyperleukocytosis (N = 270)	50	**18.5**	5	**11.4**	16	**20.5**	8	**19.1**	8	**20.0**	9	**16.4**	26	**29.2** **	11	**24.4**
	Hyponatremia (N = 203)	58	**28.6**	11	**32.4**	18	**29.0**	5	**15.2**	11	**34.4**	15	**41.7**	20	**26.3**	9	**28.1**
	Hypokalemia (N = 179)	58	**32.4**	13	**44.8**	13	**24.1**	9	**29.0**	15	**50.0** *	9	**29.0**	23	**34.3**	7	**25.9**
	Positive urine dipstick (N = 257)	50	**19.5**	13	**29.6**	13	**17.3**	2	**6.5**	24	**53.3** ***	12	**24.5**	21	**23.3**	4	**9.5**
**Clinical diagnoses cited on admission**																	
	Gastroenteritis	154	**49.5**	23	**45.1**	35	**38.9**	11	**25.0**	31	**64.6**	51	**82.3**	52	**46.9**	23	**45.1**
	Respiratory tract infection	108	**34.7**	14	**27.5**	50	**55.6**	14	**31.8**	13	**27.1**	12	**19.4**	43	**38.7**	11	**21.6**
	Malaria	59	**19.0**	10	**19.6**	17	**18.9**	39	**88.6**	3	**6.3**	14	**22.6**	28	**25.2**	4	**7.8**
	Sepsis	35	**11.3**	4	**7.8**	6	**6.7**	1	**2.3**	7	**14.6**	9	**14.5**	8	**7.2**	8	**15.7**
	Urinary tract infection	5	**1.6**	2	**3.9**	1	**1.1**	1	**2.3**	1	**2.1**	0	**0.0**	2	**1.8**	1	**2.0**

RTI: X-ray proven respiratory tract infection; Malaria: smear-microscopy proven malaria; UTI: culture and cytology-proven urinary tract infection; Infectious diarrhea: diarrhea with microbiologically proven bacterial or parasitic infection or rotavirus infection; NA: not applicable (sign included or excluded from category definition).

* p-value (compared to children without this type of infection) <0.05.

** p-value <0.01.

*** p-value <0.001.

At least one hemoglobin S allele was present in 60 of 278 children tested (22%), and 16 of these 60 children were homozygous, representing a 6% prevalence of sickle-cell disease. Among the 215 children whose parent or guardian agreed to an HIV test, 3 (1.4%) were found to be positive.

All but one child received at least one antibiotic during hospitalization, and 65 children (20.9%) received 3 or 4 different antibiotics. Ceftriaxone was used most frequently (n = 236/311, 75.9%), followed by amoxicillin (n = 121, 38.9%), amoxicillin-clavulanic acid (n = 83, 26.7%), ciprofloxacin (n = 74, 23.8%) and cloxacillin (n = 52, 16.7%).

### Bacteremia

Of 302/311 (97%) children from whom a blood specimen for culture could be obtained, growth was detected in at least one blood culture in 160 (53%), of which 89 (56%) were considered as contaminated. A pathogen was detected in 51 (17%); 4 of these children had mixed infections. The majority of organisms identified were Gram-negative bacilli, most frequently *Salmonella* spp., followed by Gram-positive cocci: *Staphylococcus aureus* (Table 2). Another 20 children were infected by possible pathogens, including *Leuconostoc* spp. (11 patients).

**Table 2:** Bacteria isolated from blood culture (N = 302), globally and in children with symptoms of SIRS on admission.

		Total (N = 302)		SIRS (N = 144)	
		n	%	n	%
**Gram negative**		**35**	**11.6**	**19**	**13.2**
	*Salmonella* spp.*	14	4.6	4	2.8
	*Salmonella* Typhi	5	1.7	4	2.8
	*Escherichia coli* *	6	2.0	5	3.5
	*Klebsiella pneumoniae* *	6	2.0	4	2.8
	*Citrobacter freundii* *	1	0.3	1	0.7
	*Haemophilus influenza*	2	0.7	1	0.7
	*Pseudomonas aeruginosa*	1	0.3	0	0.0
**Gram positive**		**21**	**7.0**	**11**	**7.6**
	*Staphylococcus aureus* *	17	5.6	8	5.6
	*Streptococcus pneumoniae* *	3	1.0	2	1.4
	*Streptococcus pyogenes*	1	0.3	1	0.7
**Possible pathogens**		**23**	**7.6**	**12**	**8.3**
	*Leuconostoc*	11	3.6	5	3.5
	*Enterococcus faecium*	4	1.3	3	2.1
	*Enterococcus faecalis*	2	0.7	0	0.0
	*Gemella morbillum* †	2	0.7	1	0.7
	*Staphylococcus epidermidis* †	1	0.3	1	0.7
	*Streptococcus equinus*	1	0.3	1	0.7
	*Streptococcus infantarius*	1	0.3	1	0.7
	*Streptococcus mutans* †	1	0.3	0	0.0

* Pathogens found in co-infections: *E. coli+C. freundii*; *E. coli+K. pneumoniae*; *S. aureus+K. pneumoniae*; *S. aureus+Salmonella* spp.; *S. aureus+S. pneumoniae*.

† Isolated in two blood cultures from the same patient.

Fever was detected in less than around a quarter of all children with a positive blood culture, and SIRS in just over half of them (Table 1). Signs of SIRS were generally present in children infected with Gram-negative organisms, except for non-typhoid salmonellae, and in only half the children with *S. aureus* bacteremia (Table 2).

### Respiratory Tract Infections

Chest X-ray was done in 291 (94%) children and 249 had a definitive interpretation by a team of pediatric radiologists in France. X-rays were found abnormal with alveolar consolidation in 90/249 cases (36.0%), in association with pleural effusion in 16 (6.4%), images suggestive of staphylococcal pneumonia (pneumatocoele or pneumothorax with effusion [17]) in 3 (1.2%), and pneumothorax in 1 (0.4%). Thirteen children with abnormal X-rays had a positive blood culture, with *Salmonella* spp. (4), *S. aureus* (3), *E. coli* (2), S. *pneumoniae, K. pneumoniae, S. pyogenes* and *C. freundii* (1 each).

Among children with respiratory symptoms on admission, 53% (n = 52/98) had abnormal X-rays, as did 25% (n = 38/152) of children with no respiratory symptoms, showing low correlation between clinical signs and X-ray results (kappa = 0.28). Fast breathing was seen in only 55.6% of children with an abnormal X-ray (Table 1).

Tuberculosis was cited as a diagnosis at admission in four children, based mostly on clinical signs. Only one of them had a chest X-ray interpreted on site as suggestive of tuberculosis, but not confirmed by expert reading in Paris. Among these four children, 2 died with tuberculosis as the main reported cause of death.

Nasal swabs from 76 randomly selected children were tested for influenza and RSV. Among those, A(H3) influenza virus was identified in 2 patients (3%), both of whom had respiratory symptoms and one of whom died with a main cause of respiratory infection. Type B RSV virus was detected in 3 children (5%), two with respiratory symptoms.

### Malaria

Out of 287 (92%) children for whom a blood smear was done, 44 cases (15.3%) of malaria infection, with *P. falciparum* in 43 and *P. malariae* in 1 case, were confirmed by smear microscopy. Only 10 (22.7%) children with microscopy-proven malaria presented with fever at admission (Table 1). Severe anemia was detected in 13 (32.5%) children with smear microscopy-confirmed malaria.

### Urinary Tract Infection

Out of 300 (96%) children tested, a urinary tract infection (UTI) was detected in 48 (16%). *E. coli* represented more than three-quarters (n = 37/48) of the microorganisms isolated, followed by *K. pneumoniae* (7), *Proteus mirabilis* (2), *P. penneri* (1) and *E. faecium* (1).

UTIs were more frequent in children under 1 year of age (24%); there was no significant association with sex (Table 1). About one-sixth of children with UTI had fever (Table 1). The urinary dipstick was positive for leukocytes and/or nitrites in around half of those with biologically confirmed UTI. Only five children (2%) in the study had an initial diagnosis of UTI, while almost two third of the children with a biologically confirmed UTI had gastroenteritis diagnosed on admission (Table 1).

### Intestinal Infection

Overall, 167 (53%) children had watery diarrhea and 10 (3%) had bloody diarrhea. An enteric pathogen was detected in 62 (37%) of children with diarrhea (Table 3). *Salmonella* spp., *C. jejuni* and all parasites were found in similar proportions among children with and without diarrhea and only *Shigella* spp. and rotavirus infections were statistically associated to diarrhea (p = 0.040 and p = 0.037 respectively). Among *Shigella* spp., *Shigella flexneri* was the most frequent (15), followed by *S. dysenteriae* (6), *S. boydii* and *S. sonnei* (2 each). Infection with *Shigella* spp. was more frequent among older children and represented 28% of diarrhea causes in children over 2 years of age. Inversely, rotavirus was more frequent among younger children, representing 17% of the cause of diarrhea in children 6 to 12 months old.

**Table 3:** Enteric pathogens isolated from stool in children with or without diarrhea (N = 307).

		Diarrhea (N = 177)		No diarrhea (N = 130)	
		n	%	n	%
**Bacteria**		**36**	**20.3**	**21**	**16.2**
	*Salmonella* spp.	18	10.2	12	9.2
	*Shigella* spp.	20	11.3	6	4.6
	*Campylobacter jejuni*	2	1.1	1	0.8
**Virus**		**23**	**13.5**	**5**	**3.8**
	Rotavirus	17	9.9	4	3.1
	Adenovirus	7	4.1	1	0.8
**Parasites**		**6**	**3.5**	**5**	**3.8**
	*Trichomonas intestinalis*	5	2.9	2	1.5
	*Entamoeba histolytica*	1	0.6	1	0.8
	*Giardia intestinalis*	0	0	2	1.5

### Infections of the Central Nervous System

Of 3 patients with suspicion of meningitis and a lumbar puncture performed, one was positive for *H. influenzae* type b, which was also isolated from this patient’s blood culture.

### Sepsis

An infection was biologically confirmed or suspected based on X-ray results in 111 of 161 children with SIRS, thus identifying sepsis in 35.7% of the study population. Sepsis was cited as a diagnosis on admission in only 8 children with confirmed sepsis (7%, Table 1).

### No Laboratory or X-ray Confirmed Infection

Eighty-nine (28.6%) patients had no laboratory or radiography-confirmed diagnosis. Of these, 38 had at least one examination (mostly chest X-ray) missing, leaving 51 patients with all systematic laboratory and radiography tests performed and no confirmed infection. The only statistically significant differences between this group and other children with a laboratory or radiography confirmed infection were a lower proportion of children with fast breathing and a higher proportion of sickle-cell disease.

### Antibiotic Resistance

Most enterobacteriacea isolated in this study were resistant to amoxicillin and cotrimoxazole but susceptible to ceftazidime/ceftriaxone, gentamicin and quinolones (Table 4). Among the organisms isolated from urine (*E. coli* and *K. pneumoniae*), almost 20% were resistant to gentamicin and almost half to cefalotine. All three specimens with extended-spectrum beta-lactamase (ESBL) found in this study (two *E. coli* and one *K. pneumoniae*) were isolated from urine. Almost all *S. aureus* were resistant to penicillin G (94%, n = 16/17) but none to cloxacillin. All three isolates of *S. pneumoniae* tested were sensitive to beta-lactams. Additional details on antibiotic resistance are published elsewhere [22].

**Table 4:** Proportion of antibiotic-resistant isolates of enterobacteriacae by bacterial genre.

	*Salmonella* spp (N = 44)		*Shigella* spp. (N = 26)		*E.coli* (N = 41)		*Klebsiella* spp. (N = 12)	
	n	%	n	%	n	%	n	%
Amoxicillin	23	**52**	19	**73**	41	**100**	12	**100**
Amoxi-clavulanic acid	9	**21**	14	**54**	25	**61**	7	**58**
Cefalotine	3	**7**	1	**4**	18	**44**	5	**42**
Cefoxitine	0	**0**	0	**0**	2	**5**	1	**8**
Cefotaxime	0	**0**	0	**0**	2	**5**	1	**8**
Ceftazidime	0	**0**	0	**0**	2	**5**	1	**8**
Imipeneme	0	**0**	0	**0**	0	**0**	0	**0**
Gentamicin	0	**0**	0	**0**	4	**10**	5	**42**
Amikacin	0	**0**	1	**4**	0	**0**	0	**0**
Nalidixic acid	2	**5**	2	**8**	5	**12**	0	**0**
Ofloxacin	2	**5**	2	**8**	4	**10**	0	**0**
Cotrimoxazole	21	**48**	24	**92**	39	**95**	8	**67**
ESBL	0	**0**	0	**0**	2	**5**	1	**8**

### Outcomes

The median length of stay in the ITFC was 8 days (IQR: 6–13 days). Twenty-nine children (9%) died. Almost half of all deaths (48%, n = 14/29) occurred within 48 h of admission. The main causes of death recorded were sepsis (15), respiratory tract infection (4), and clinical suspicion of tuberculosis (2). Overall, 20 (69%) children who died had one or several laboratory or X-ray proven infections, including 8 bacteremia (4 *S. aureus*, 2. *H. influenzae*, 1 *Salmonella* spp., 1 *E. coli*); 7 UTI (6 *E. coli*, 1 *K. pneumoniae*); 3 infectious diarrhea (1 *S. flexneri*, 1 *S. sonnei*, 1 *Salmonella* spp.); 2 malaria; and 2 RTI. Of the nine children who died with no confirmed infection, 6 did not have a chest X-ray. For these, the main cause of death reported by the clinicians was respiratory tract infection in 3, sepsis in 2, and measles in one child. In the 3 children with all examinations performed and no confirmed infection, the main cause of death was sepsis in 2 children and anemia in 1 child. The case fatality ratio (CFR) was not significantly different between children with a proven infection (9%) or with no proven infection (10%, p = 0.76).

The CFR was 16% (n = 8/51, p = 0.1) among patients with a positive blood culture, 15% (n = 7/41, p = 0.2) among children with a UTI, 8.3% (n = 4/62, p = 0.5) among children with infectious diarrhea, and 4.5% (n = 2/44, p = 0.5) among those with malaria. Since X-ray could not be performed due to their severe condition on admission in 18 of the 29 children who subsequently died, we considered that the CFR among radiography-confirmed respiratory tract infections would be biased and not representative of all respiratory tract infections.

The only clinical signs associated with death were lethargy (17%, 18/108, p = 0.001), severe dehydration (CFR 36%, n = 4/11, p = 0.002), and hepatomegaly (CFR 23%, n = 8/35, p = 0.03).

## Discussion

The findings of this clinical and biological description of infections among severely malnourished children underline the importance of bacterial and other infections in these vulnerable children and the low value of clinical signs for their diagnosis. These findings provide preliminary data for developing more evidence-based strategies to treat infections in this population.

In our cohort, gastroenteritis, respiratory infections and malaria were the most frequent main clinical diagnoses on admission, based mostly on clinical signs and the few biological tests available immediately to clinicians, i.e. hemoglobin and malaria RDTs. Overall, a bacterial, viral or parasitic agent was identified in over half of the children; and about one-third of study participants had septic condition. Radiography and laboratory results confirmed the high prevalence of respiratory infections and the importance of bacteremia and UTI, present in 17% and 16%, respectively, and largely under-diagnosed on admission. The proportion of positive blood cultures (17%) is similar to that found among hospitalized children with complicated SAM in Uganda and Kenya [23], [24] but higher than the 10% reported elsewhere [25]–[27].

Clinical signs at inclusion, such as fever for bloodstream infection or malaria, or WHO-recommended guidelines for clinical diagnosis of pneumonia proved to be poor indicators of infections. This observation is consistent with other published findings showing that these criteria are far less reliable among malnourished children [28]. Consequently, the diagnoses on admission often did not correlate with the laboratory- or radiography-confirmed diagnosis, except for malaria, for which rapid tests are available.

As in other parts of the world, *E. coli* was the main cause of UTI. *S. aureus* and non-typhoidal *Salmonella* were the most common pathogens detected in blood cultures. Non-typhoidal salmonellae have been consistently described as an important cause of invasive disease in children and adults in Africa, with higher CFR than found in our study [29], [30]. In contrast, *S. aureus* is generally not the predominant Gram-positive organism found in African children [31], and children with SAM might be more at risk for infection with *S. aureus* [23]. Considering that neither the Hib nor pneumococcal vaccines had been introduced in Niger at the time of this study, the proportion of infections with these pathogens was low compared to other settings [31]. Whether this reflects a low sensitivity of blood culture or a true low prevalence of *S. pneumoniae,* as reported elsewhere [32], [33], remains to be confirmed. In addition, several atypical bacteria were isolated from blood, for which pathogenicity could not be established formally, considering the high level of contamination of blood culture in the study. The most frequent was *Leuconostoc*, which has been described as a cause of sepsis in malnourished, immunocompromised patients, or patients with disrupted bowel mucosa [34], [35], and should be investigated further.

The enterobacteria isolated in the study showed a significant level of resistance to amoxicillin and cotrimoxazole but were mostly sensitive to third-generation cephalosporins, quinolones, macrolides and aminoglycosides, consistent with previous findings in Africa [31], [36]. The low sensitivity to amoxicillin is concerning, since this drug is the routine systematic treatment for severely malnourished children without other severe conditions [5]. The high level of intestinal carriage of ESBL bacteria on admission in our population, shown in a sub-study [22], did not translate into similar levels of ESBL bacterial infections. However, three children had a UTI caused by ESBL bacteria, which were resistant to most of the antibiotics available in Niger. This, together with the high rate of ESBL bacteria acquisition during hospitalization at the ITFC [22], highlights the need to closely monitor antibiotic resistance.

Our study has several limitations. First, a sample size of 1000 children over a period of at least one year was initially judged necessary to estimate the case fraction of different types of infection and to document antibiotic resistance of the main pathogens isolated. We did not reach this target sample size due to the premature closure of the MSF program and as a result, we did not recruit patients for an entire calendar year, missing in particular the months of August to October, which correspond to the malnutrition and malaria peaks. Second, despite rigorous standardized clinical and laboratory assessments, the diagnostic arsenal was still limited compared to developed country settings and it was difficult to ascertain diagnoses in some cases. In particular, diagnosis of tuberculosis was based only on clinical signs. Third, glycemia, which is an important indicator of infection and severity, was not analyzed here because it was measured after children were tested for anorexia by giving them therapeutic food, which limited its interpretation. Fourth, blood culture contamination probably interfered with our ability to isolate pathogens, and reflects the extreme difficulty of maintaining high-quality laboratory practices, both at bedside and in the laboratory, in resource-limited settings. Finally, the burden of infectious diseases overall and particularly among children who died might have been underestimated as a result of the general lack of sensitivity of blood culture, contaminations in these, as well as the fact that around one fifth of the children did not have a definitive radiography result.

In conclusion, our study confirms the high level of infections among severely ill children with SAM and that diagnosis based only on clinical signs and isolated rapid tests is poorly indicative of the type of infection in this population. Although in agreement with the current recommendations to use broad-spectrum antibiotics as first-line treatment, these results contribute rare data from the Sahel to the ongoing debate on the choice of antibiotherapy between the currently WHO-recommended ampicillin-gentamicin combination and a third generation cephalosporin [29]. Considering (i) the high resistance levels of enterobacteriae to ampicillin and, to some extent, to gentamicin; (ii) the ease of ceftriaxone delivery (a once-daily IM injection); (iii) and the relatively low mortality in our cohort compared with other settings [6], ceftriaxone may be an appropriate alternative. However, this must be balanced with the worrying emergence and acquisition of ESBL strains in these settings [22]. Our results reinforce current calls to implement robust strategies for limiting the emergence of antibiotic-resistant bacterial strains including: better diagnostic capacities and the development of field-adapted diagnostic tests; reinforcing infection control practices, such as strict hand hygiene; and reinforcing regional antimicrobial resistance surveillance networks, such as those established for specific bacteria or other pathogens, e.g. netSPEAR or WWARN. Furthermore, expanding both descriptive and interventional studies on the treatment of malnourished populations is essential for addressing the needs of this vulnerable population.

## Supporting Information

Table S1 (DOCX)
