# Supplementary material for: Infections in Children Admitted with Complicated Severe Acute Malnutrition in Niger
Source: PLoS One. 2013 Jul 17;8(7):e68699. doi: 10.1371/journal.pone.0068699 (PMC3714292; doi:10.1371/journal.pone.0068699)
Supplement: Table S1 — (DOCX) [file pone.0068699.s001.docx]

Table S1. Main socio-demographic and nutritional characteristics of the study population and other children admitted directly to the ITFC during the study period

|  | Study (N=311) | | Not in study (N=2,256) | | p-value |
| --- | --- | --- | --- | --- | --- |
|  | n | % | n | % |  |
| Sex (male) | 170 | 54.7 | 1275 | 56.5 | 0.537 |
| Age - median [IQR] | 13 | [10-24] | 13 | [10-22] | 0.862 |
| 6 to 11 months | 127 | 40.8 | 811 | 36.0 |  |
| 12 to 23 months | 100 | 32.2 | 899 | 39.9 |  |
| 24 to 36 months | 63 | 20.3 | 404 | 17.9 |  |
| 37 to 59 months | 21 | 6.8 | 125 | 5.5 |  |
| Origin (Nigeria) | 72 | 23.2 | 537 | 23.8 | 0.592 |
| MUAC median [IQR] | 116 | [108-122] | 118 | [110-124] | **0.048** |
| Oedematous malnutrition | 48 | 15.4 | 309 | 13.7 | 0.407 |
| WfH z-score median [IQR] | -3.8 | [-3.3:-4.5] | -3.8 | [-3.3;-4.6] | 0.714 |
